# Efficient radiative cooling of low-cost BaSO_4_ paint-paper dual-layer thin films

**DOI:** 10.1515/nanoph-2023-0642

**Published:** 2024-01-23

**Authors:** Andrea Felicelli, Jie Wang, Dudong Feng, Endrina Forti, Sami El Awad Azrak, Joseph Peoples, Jeffrey Youngblood, George Chiu, Xiulin Ruan

**Affiliations:** School of Mechanical Engineering and Birck Nanotechnology Center, Purdue University, West Lafayette, IN, USA

**Keywords:** radiative cooling, daytime cooling, nanocellulose, environmental impact, nanoparticle coatings, nanoscale energy

## Abstract

Many materials have been explored for the purpose of creating structures with high radiative cooling potential, such as nanocellulose-based structures and nanoparticle-based coatings, which have been reported with environmentally friendly attributes and high solar reflectance in current literature. They each have their own advantages and disadvantages in practice. It is worth noting that nanocellulose-based structures have an absorption peak in the UV wavelengths, which results in a lower total solar reflectance and, consequently, reduce radiative cooling capabilities. However, the interwoven-fiber structure of cellulose gives high mechanical strength, which promotes its application in different scenarios. The application of nanoplatelet-based coatings is limited due to the need for high volume of nanoparticles to reach their signature high solar reflectance. This requirement weakens the polymer matrix and results in more brittle structures. This work proposes a dual-layer system, comprising of a cellulose-based substrate as the bottom layer and a thin nanoparticle-based radiative cooling paint as the top layer, where both radiative cooling potential and mechanical strength can be maximized. Experimental and theoretical studies are conducted to investigate the relationship between thickness and reflectance in the top coating layer with a consistent thickness of the bottom layer. The saturation point is identified in this relationship and used to determine the optimal thickness for the top-layer to maximize material use efficiency. With the use of cotton paper painted with a 125 μm BaSO_4_-based layer, the cooling performance is enhanced to be 149.6 W/m^2^ achieved by the improved total solar reflectance from 80 % to 93 %.

## 1 Introduction

In 2019, the World Economic Forum reported that greenhouse gas emissions from air conditioning technologies will account for as much as a 0.5 °C increase in global temperatures by the end of the century [1]. Moreover, these traditional air conditioning technologies not only contribute to global warming but are out of reach for up to 92 % of the world’s population. Extreme heat conditions are predicted to result in annual loss of 255,000 lives by 2050 according to the World Health Organization [2]. As sustainable air conditioning has clearly acquired a crucial role in fighting climate change and its consequences, radiative cooling – a highly promising passive cooling phenomenon – has been chosen to be a preferred foundation for developing state-of-the-art air conditioning alternative technologies in the past few decades due to its versatility, environmental friendliness, and accessibility. By utilizing the large temperature differential between the frigid deep outer space and the 300 K surface of the earth, radiative cooling facilitates energy transfer [3]. By harnessing the atmospherically transparent “sky window” in the range of 8–13 μm, up to 150 W/m^2^ of thermal energy can be emitted into deep space and another 1000 W/m^2^ of solar irradiation reaching the Earth’s surface can be reflected [4]. This results in a cooling effect on the Earth’s surface.

Radiative cooling technologies have been employed in a wide range of applications with various forms, including paintable technologies [5]–[10], integrated photonic structures [4], [11]–[14], metal–polymer composites [15], [16], and dielectric metamaterials [17]–[19]. Each form has its unique features in different functionalities – for example, paintable technologies provide cooling solutions that are versatile and easy to apply, while composite materials can enhance mechanical robustness. While many innovations and advances have already been seen in technologies for radiative cooling applications, multifunctionality and accessibility of these technologies are the two critical factors that require further development. For example, nanoparticle-based paintable coatings have achieved high solar reflectance based on layer thickness reported in current literature [6], [7], [9]; however, a high volume concentration of filler materials such as CaCO_3_, BaSO_4_, and h-BN, as well as a coating thickness that exceeds the optimal value is often needed to achieve high solar reflectance, resulting in increased costs. Some other materials, such as cellulose-based structures, are accessible and inexpensive options with desirable mechanical strength [20], [21], but their radiative cooling performance is limited by cellulose’s absorption in UV and NIR bands. Radiative cooling works have used cellulose-based, or derived, porous polymer systems to achieve solar spectrum reflectance [22], [23]. However, there is still significant absorbance in ultraviolet wavelengths that is absent in the previously mentioned cooling paints. Previously, cellulose-fiber paper and PTFE coatings were stacked to improve mechanical robustness and weatherability as well as increasing total solar reflectance [24]. However, the stacked systems come to a relatively high film thickness of 700 μm, and the effect of paint layer thickness on optical performance was not fully studied and alternative cellulose-based substrates were not considered; these factors could improve cost effectiveness and material efficiency.

To improve multifunctionality and optimize radiative cooling performance, multilayer systems are often constructed with a highly reflective top layer and a highly emissive bottom layer in recent cooling technologies. These include TiO_2_–SiO_2_ dual-layer systems with 90.11 % total solar reflectance [25], a system composed of acrylic and TiO_2_ coating on carbon black with 91 % total solar reflectance [26], and a coating composed of SiO_2_, TiO_2_, and PDMS that achieves 91 % total solar reflectance [27]. These systems effectively optimize mechanical and optical performance while maintaining a relatively low thickness, but they tend to show relatively low total solar reflectance and often have reflective layers that require specialized application techniques. This can negatively impact their cost effectiveness and practicability. Some dual-layer systems use a metallic layer to boost back reflectance of the system – for example, dual-layer systems with PTFE coated on silver were able to achieve 99.1 % total solar reflectance [28]. Unfortunately, such systems are limited in their scalability, weight, and cost-effectiveness despite delivering exceptional cooling performance and mechanical robustness.

In this study, a dual-layer system composed of a cellulose-based cotton paper as the bottom layer and a thin nanoparticle-based coating as the top layer is proposed. This system is designed to maximize radiative cooling potential while ensuring cost effectiveness and mechanical robustness. We first evaluate optical characteristics and nanostructures of different nanocellulose-based films, including visually semi-transparent and transparent pure nanocellulose films [29], [30], cotton pulp films, and cellulose acetate films. Cellulose acetate and cotton pulp paper were chosen as substrates for the dual-layer system as they demonstrated the greatest sky window emissivity and total solar reflectance. For the top layer, BaSO_4_-acrylic paints were chosen for their ease of application and high reported total solar reflectance of 98.1 % [7]. The optimal thickness of the top layer was determined by identifying the saturation point in the relationship between thickness and total solar reflectance. It turns out that the designed dual-layer film has low cost and high mechanical robustness, as well as the desired radiative cooling properties of the nanoparticle paints. This work also serves as proof-of-concept that a two-layer approach can help to combine benefits and reduce drawbacks of both components, in this case even with a very inexpensive, less weather-resistant cotton paper substrate. This approach can be applied to other substrates with similar properties – rigidity, higher than desired absorbance, lower abrasion/water resistance – to improve the reflectance while providing reinforcement of a very thin highly reflective cooling paint layer. This system provides an option to low-budget applications, and many packaging applications use paper and desire cooling, and this dual-layer design can provide the cooling benefit.

## 2 Results and discussion

**Figure 1:**
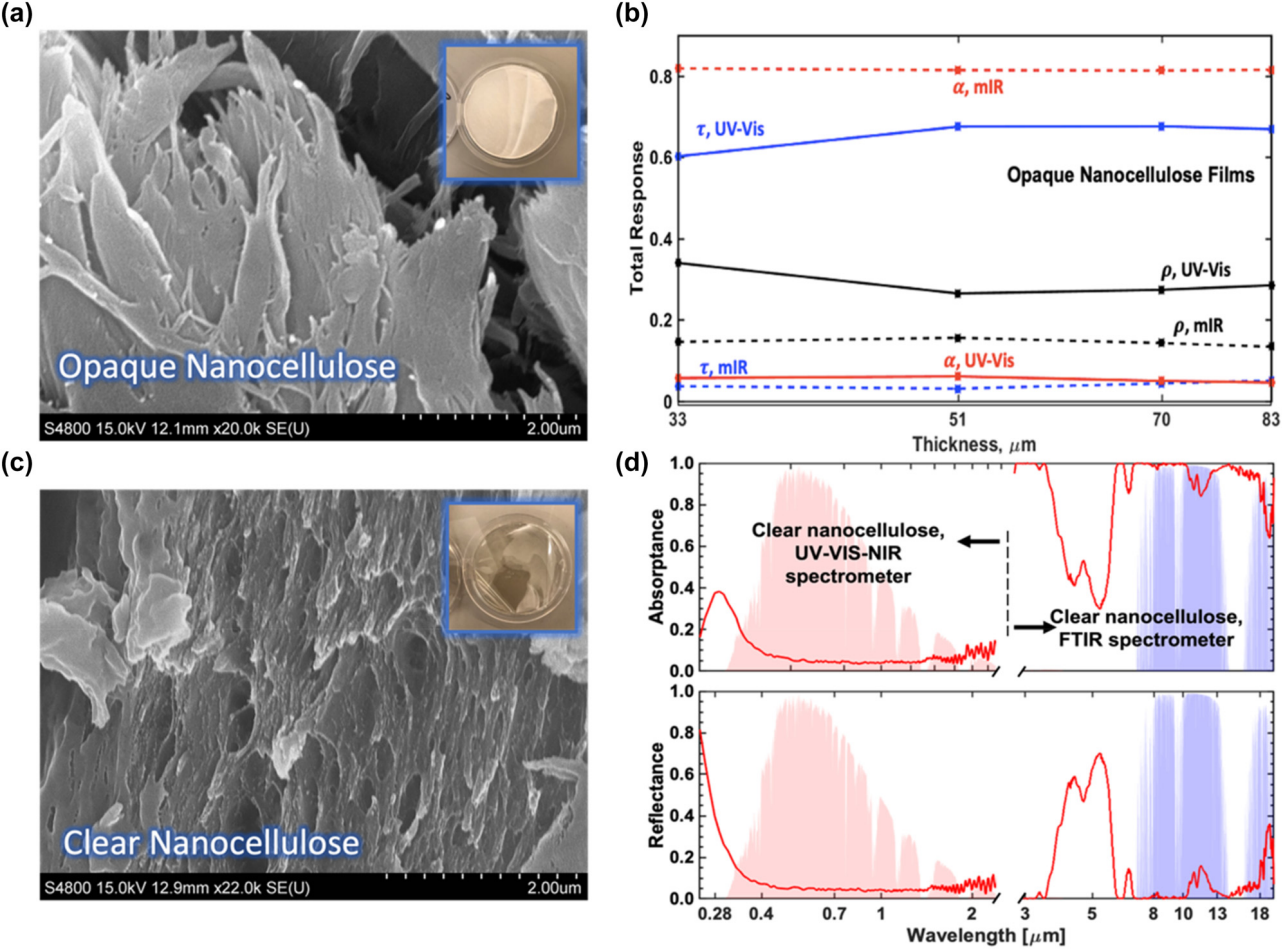
SEM and optical characterization of pure nanocellulose samples. (a) SEM image of fibers of a pure nanocellulose semi-transparent film; top right corner shows the photo of sample. (b) Total solar and sky window reflectance, transmittance, and absorptance compared as a function of semi-transparent pure nanocellulose film thickness. Thicknesses of films under test are 33, 51, 70, and 83 μm. Example full spectra is shown in Figure S1a and b of supplementary text. (c) SEM image of fibers in pure nanocellulose transparent film; top right corner shows the photo of sample. (d) Absorptance and reflectance spectra from 0.25 to 20 μm wavelengths for clear pure nanocellulose film of 35 μm thickness. The values for wavelengths from 0.25 to 2.5 μm were measured using a UV–Vis-NIR spectrometer, while those for wavelengths from 3 to 20 μm were measured using an FT-IR spectrometer. The same sample was used for all measurements.

Nanocellulose has been found to be a versatile material with varying optical properties depending on its nanostructure. For example, the fiber scale can be manipulated to reduce or increase visible light transmission. Two forms of pure nanocellulose were studied to determine their solar spectrum reflectance and sky window emissivity, as well as the correlation between their nanostructure and associated optical characteristics. The first form is a visibly semi-transparent cellulose nanofibril sheet [29], with smaller fibers on the scale of several hundred nanometers to a few microns (Figure 1a). Four thicknesses were characterized for this film at 33, 51, 70, and 83 μm. These thicknesses show little variations in the total solar absorptance, transmittance, and reflectance or in the sky window emissivity (Figure 1b). Keeping in mind the high amount of transmission for films of thicknesses less than 100 μm, the total solar reflectance was observed to be 35–40 %. However, it is worth noting that the total solar absorptance was considerable for this thickness at 7–10 %, which would likely increase with thicker films. It confirms that nontrivial absorptance is often seen in cellulose-based materials in the ultraviolet (UV) and near-infrared (NIR) regions of the solar spectrum wavelengths.

Visibly clear nanocellulose sheets, composed of cellulose nanofibril epoxy laminates [30], was found when imaged under SEM to have a smaller fiber scale and presence of larger pores; this allows for the visible light transmission (Figure 1c). For this film, one thickness of 35 μm was characterized optically. The film was found to have relatively low absorption in the solar spectrum, with the majority of the absorption present in the ultraviolet wavelengths. Interestingly, the sky window emissivity was found to be a high 0.97, confirming nanocellulose to be an effective emitter in these wavelengths (Figure 1d).

**Figure 2:**
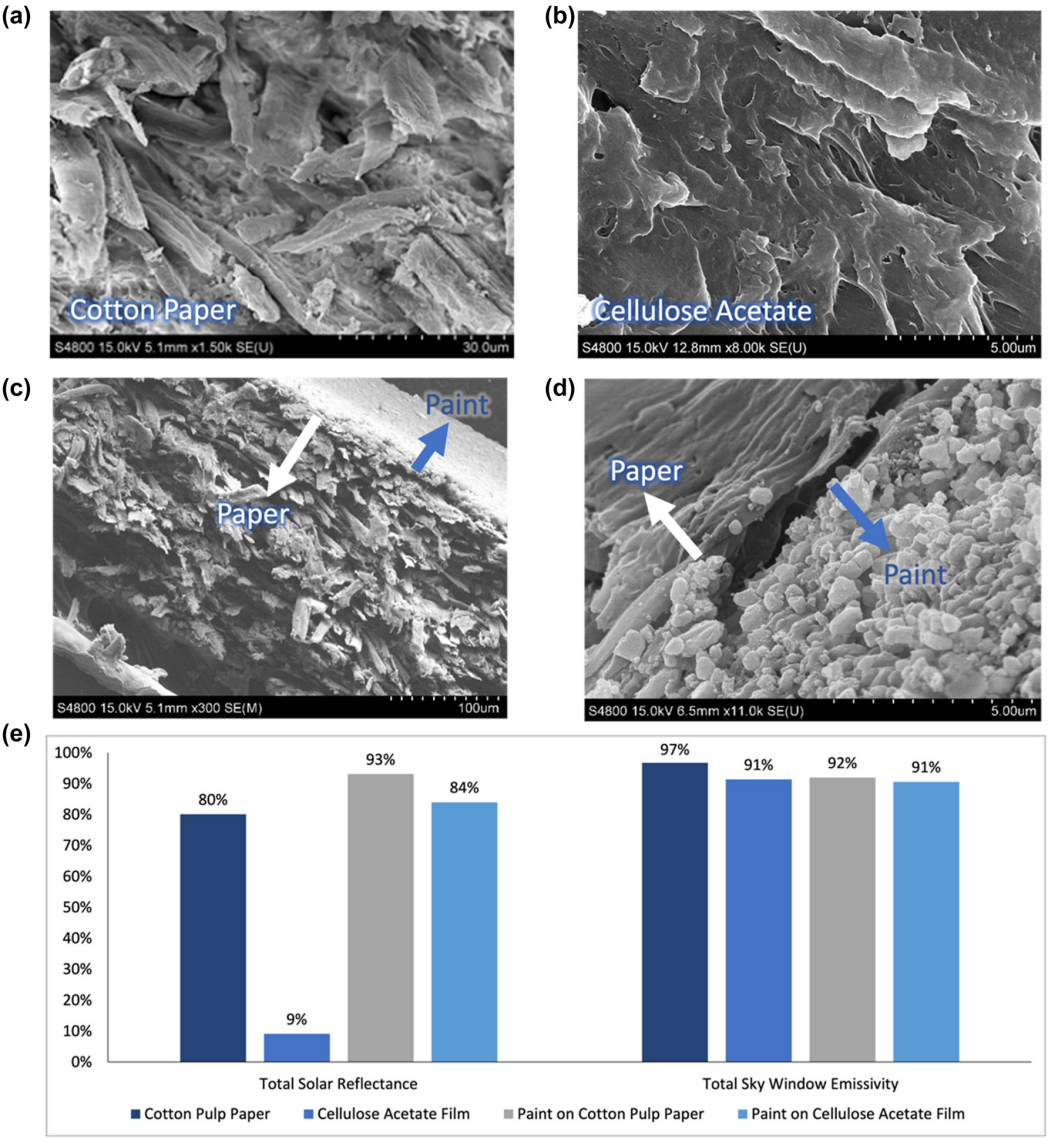
SEM and spectral characterization of optical properties for dual-layer films and other forms of cellulose. (a) SEM image of fibers in pure cotton pulp paper. (b) SEM image of fibers in cellulose acetate film. (c) SEM image of dual-layer films showing cross-sectional view of cotton paper fibers. (d) SEM image of dual-layer films showing intersection and paint structure. (e) Total solar reflectance and sky window emissivity comparison of dual-layer films made on cotton paper and cellulose acetate. Full example spectra for paint on cotton pulp paper can be found in Figure 2a and b of supplementary material.

Another substrate, cotton-fiber paper is a visually white, cellulose-based material made from 100 % cotton pulp. When magnified using SEM imaging, the structure of the material is seen to be made of small-scale fibers in randomized orientations, with a small amount of porosity in the form of air pockets (Figure 2a). Measuring the optical properties of this commercial cotton paper gave a solar reflectance of 80 % and a sky window emissivity of 96.7 %. This radiative cooling performance is consistent with the previous prediction of effective light-scattering based on the microstructure. However, as with other cellulose structures, the considerable absorptance in the ultraviolet as well as a small amount in the near-infrared wavelengths lead to a lower solar reflectance value than is ideal when compared to the 98.1 % reflectance of BaSO_4_-acryclic paint. Another drawback is the less-than-ideal resilience of a paper material against the elements when used in outdoor applications, where radiative cooling technology is most needed – paper materials are known to be susceptible to water damage as well as having low abrasion resistance. However, the rigidity of the paper provides more mechanical robustness than a coating on its own, allowing for a thinner coating of paint to be used. This is also true for cellulose acetate, another visibly transparent cellulose-based substrate with mechanical rigidity and less permeability to water than cotton pulp due to its film-like surface. When measured, cellulose acetate shows similarly high sky window emissivity of 91 % and high transparency in the solar spectrum. This makes it a good substrate to compare with cotton pulp paper in order to separate the effects of the original substrate solar reflectance and the improvement from an added paint layer at the bottom. When looked at under SEM, cellulose acetate shows higher porosity and a smaller scale of cellulose fibers that allow for visible light transmission (Figure 2b).

Despite the mentioned mechanical limitations, the radiative cooling performance of cotton paper is a good baseline, and one that can be improved when used in conjunction with a thin layer of coating. When used on its own, barium sulfate–based radiative cooling paints need a layer thickness of about 400 μm in order to achieve a high reflectance of 98 % [7]. However, when the paint is evenly coated on cotton paper, the two materials could work together to demonstrate a comparably high solar reflectance while requiring a much thinner layer of paint. To test this, we created a multilayer film with a 125 μm layer of paint, consisting of 60 % concentration barium sulfate nanoparticles in an acrylic matrix, coated evenly over the commercial white cotton paper. We confirmed even distribution of the coating via SEM imaging, which also demonstrated the difference between the structures of the materials (Figure 2c and d).

Optical measurements were also performed on the multilayered film. With a 125 μm layer of BaSO_4_-based paint, we were able to increase the solar reflectance to 93 %. This is a 13 % increase in solar reflectance compared to the cotton paper on its own, which accounts for an extra 149.6 W/m^2^ in the multilayered film from reflected solar irradiation. A paint layer of the same thickness was also painted on the cellulose acetate sheet, improving the solar reflectance to 84 % from 9 %. This comparison between cellulose acetate, cotton paper, and dual-layer system based on both allows us to better isolate the improvements from the dual-layer system (Figure 2e).

**Figure 3:**
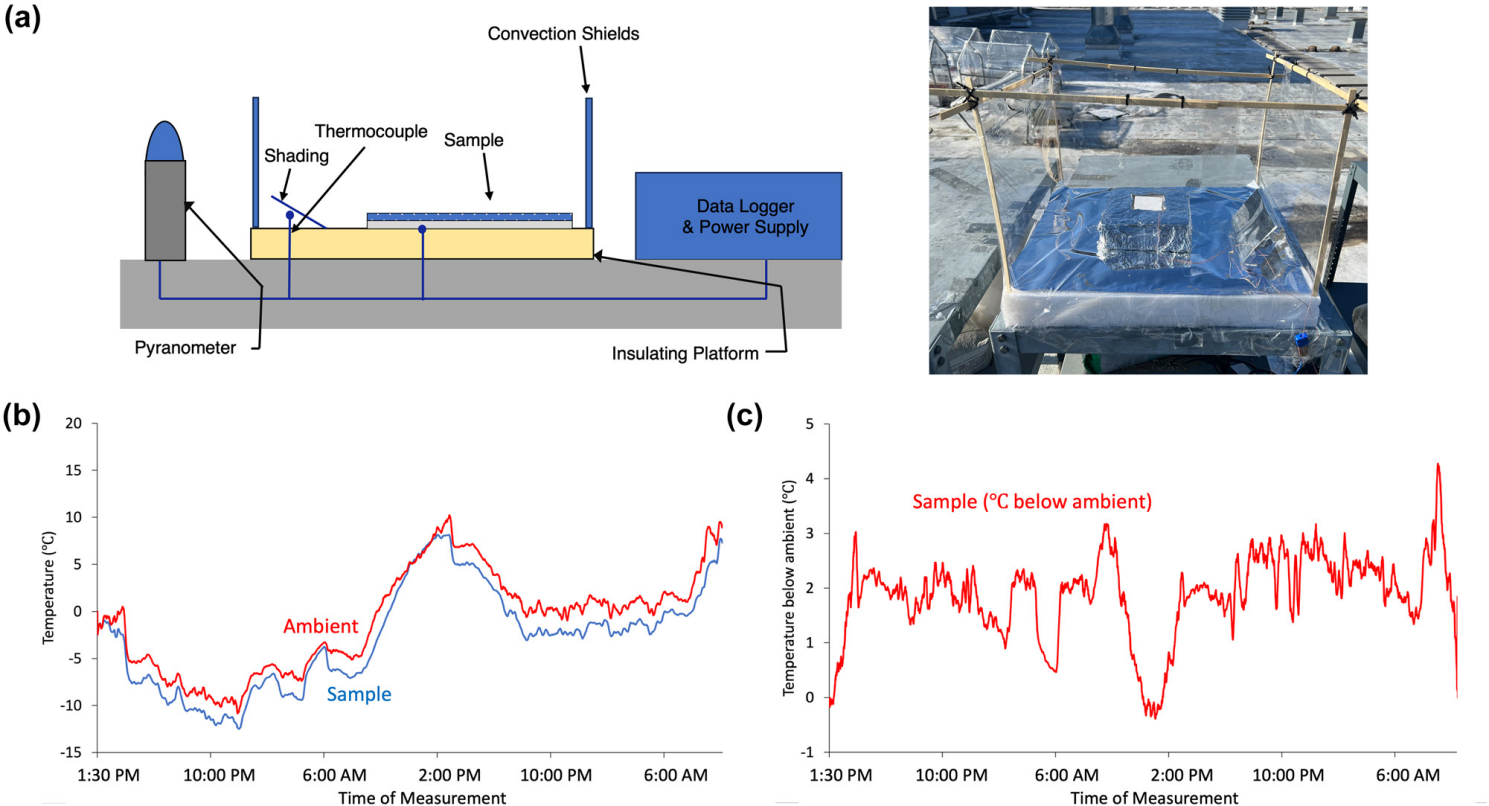
Field testing setup and results. (a) Field test setup photograph and schematic. More details are given in the Methods section. (b) Temperature measurements of both ambient and sample during the outdoor tests over two consecutive days from November 28–30, 2023. The measured average dew point for this period was −9.2 ± 5.1 °C, and relative humidity was 65.1 ± 14.9 %. The test was performed with samples with 150 μm of paint on the 1-mm cotton paper. Data demonstrate moving average over 10 min increments of the measured data. (c) Degrees Celsius below ambient measured for sample. In this graph, positive numbers denote below ambient cooling. Data demonstrate moving average over 10 min increments of the measured data.

Field tests were conducted on the dual-layer films in order to demonstrate their radiative cooling performance. The experimental setup, as illustrated in Figure 3a and detailed in the Methods section, spanned a 46-h period under mostly clear skies, with ambient temperatures ranging from −15 to 10 °C. The results, depicted in Figure 3b and c, revealed a consistent lower temperature of the measured sample compared to the ambient temperature, persisting throughout both daytime and nighttime conditions. On average, the sample temperature remained approximately 1.2 °C below ambient, highlighting the effective radiative cooling capability of the dual-layer films. The most substantial cooling effect was observed at 6 AM, registering a temperature 4 °C below ambient. In summary, these outdoor tests indicate promising radiative cooling performance of the dual-layer films, particularly emphasizing their ability to maintain lower temperatures than the surrounding environment. Such findings underscore the potential practical applications of these films in temperature regulation or cooling systems.

**Figure 4:**
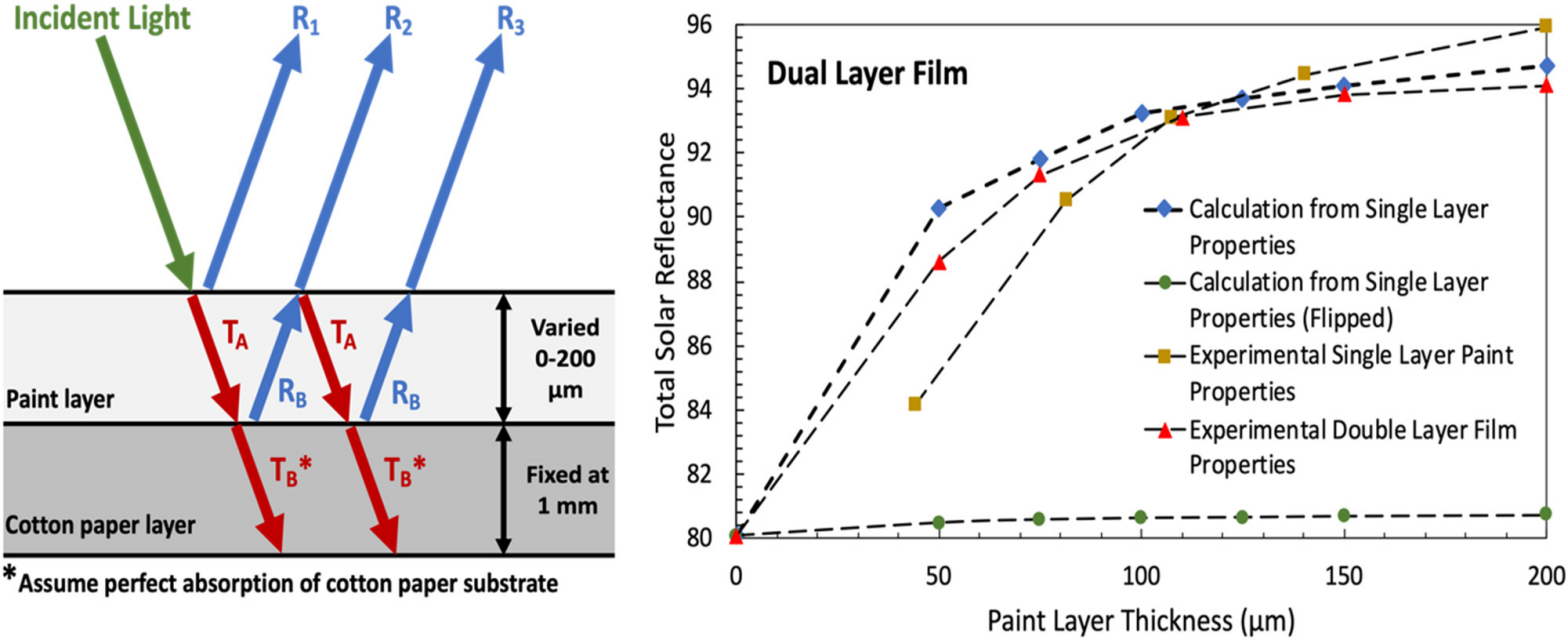
Total solar reflectance theoretical estimate schematic and results. (a) Schematic of solar reflectance estimate calculation technique. This is described in greater detail under methodology. Perfect absorbing boundary condition assumed at bottom surface of bottom layer. (b) Plot of total solar reflectance versus paint layer thickness, comparing calculated estimate for dual layer from both sides to experimental single layer paint and double layer film values. Single layer values for BaSO_4_ paints are from Li et al. [7].

To maximize material usage and cost effectiveness, a study was conducted on total solar reflectance versus paint top layer thickness on the cotton pulp paper substrate. In this study, only the thickness of the paint layer was varied from 0 to 200 μm, while the cotton paper thickness was kept consistently at 1 mm. To validate the experimental results, a total solar reflectance theoretical estimate was also calculated based on single-layer properties of the BaSO_4_-acrylic paint individually and the cotton pulp paper individually (Figure 4a). More information on this calculation can be found in the Methods section. Furthermore, the theoretical estimate was calculated for incident sunlight entering from the paint side of the dual-layer system and from the cotton paper side of the system (effectively turning it “upside down”). While theoretical and experimental results demonstrate the significant increase in total solar reflectance when a paint top layer is added in increasing thickness to the cotton pulp paper, the theoretical estimate for the reverse system shows little improvement (Figure 4b). This demonstrates the importance of putting the highly reflective and nonabsorbing layer at the top – when the transmitted light reaches the bottom layer, it is already much attenuated but with little absorption by the top layer. However, when the system is reversed, the high thickness and relatively higher absorptance in solar wavelengths by the cotton paper don’t allow for many improvements to come from the added paint layer. In this comparison, we are also able to identify 125 μm as a saturation point for the BaSO_4_ paint layer thickness, after which increasing the thickness by the same interval of 25 μm gives an increasingly lower percent return in total solar reflectance. Therefore, 125 μm is identified as an ideal thickness for this system to minimize material usage and while significantly increasing radiative cooling performance and improving cost effectiveness.

**Figure 5:**
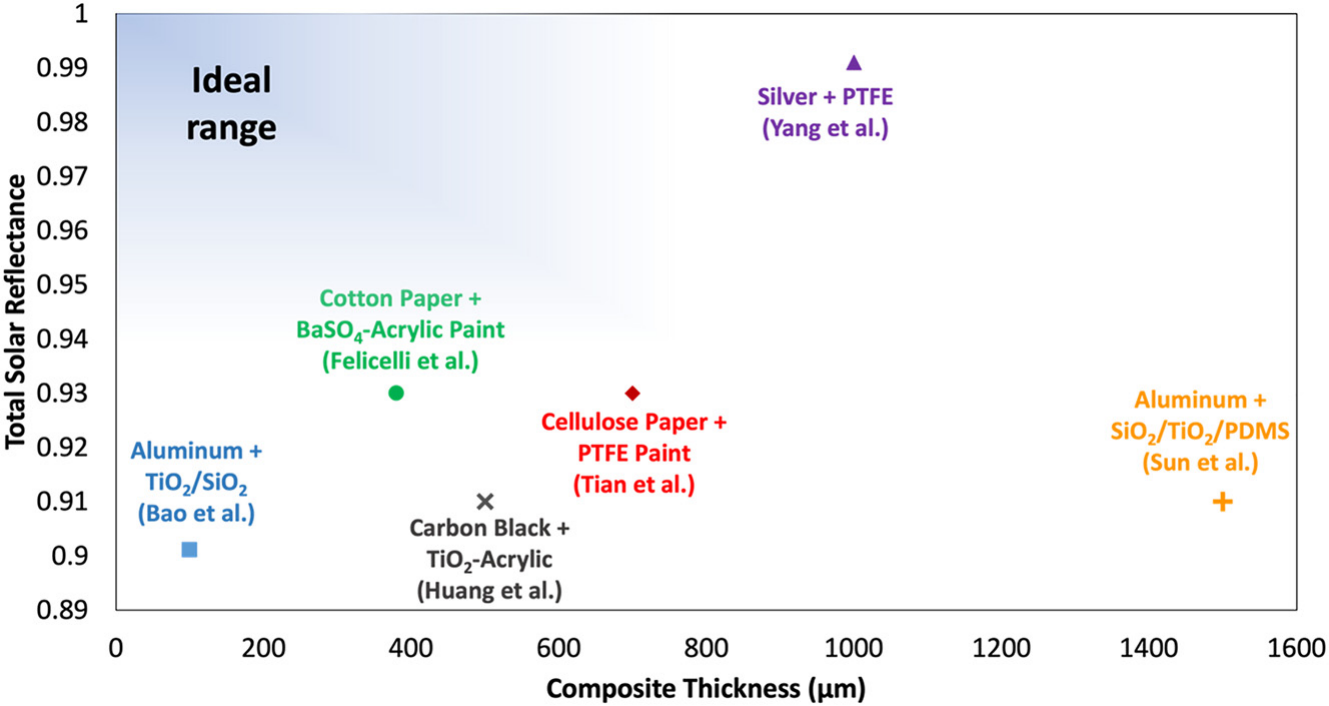
Comparison of total solar reflectance and composite thicknesses of the dual-layer films featured in this work as well as other layered composites in literature.

This reflectance versus thickness study was also used for comparison to other developed dual-layer systems [24]–[27]. With the goal of minimizing thickness while maximizing total solar reflectance, the proposed dual-layer system of BaSO_4_-acrylic paints on top of cotton pulp paper achieved better tradeoff between paint thickness and total solar reflectance as compared to other works (Figure 5).

For further comparison, as well as demonstration of radiative cooling power, a figure of merit was used for the dual-layer films in comparison with other systems. The overall radiative cooling capabilities of materials can be thus effectively compared using the previously established RC figure of merit [6]. It is defined as:

$$\text{RC}=\varepsilon_{\text{sky}}-r\left(1-R_{\text{solar}}\right)$$

Here, *ε*_sky_ represents the total sky window emissivity, *r* is the ratio of solar irradiation power to the blackbody surface emissive power in the sky window (recommended to be set around 7.14, assuming standard values of 1000 W/m^2^ for solar irradiation power and 140 W/m^2^ for blackbody surface emissive power in the sky window), and *R*_solar_ is the total solar reflectance. The meaning of the figure of merit is that multiplying it with the blackbody surface emissive power in the sky window provides the net radiative cooling power. According to this definition, a positive RC suggests the potential for cooling below ambient conditions. The significance of this figure of merit is highlighted by the fact that each 0.01 increment in solar reflectance corresponds to a 0.0714 increment in sky window emissivity in terms of cooling performance. Using this approach, the value of RC for this dual-layer system is calculated to be 0.42, which indicates ability to achieve subambient cooling. This can be compared to other systems, both paintable and composite with RC’s of 0.77 [7], 0.32 [4], 0.53 [18], 0.35 [31], and 0.57 [10]. Looking at this range, the proposed dual-layer work achieves an RC figure of merit comparable or higher than some other systems in literature, with the bonus of being inexpensive.

To summarize, in this work, a dual-layer system was developed to improve cost effectiveness, mechanical rigidity, material use, and radiative cooling performance. This was accomplished in the form of coating a thin layer of BaSO_4_-acrylic radiative cooling paint on top of cotton pulp paper, which was able to improve the performance of the moderately reflective and mechanically sturdier cellulose-based substrate by increasing the total solar reflectance from 80 % for the cotton paper to 93 % with only 125 μm of paint top layer. In terms of RC, the radiative cooling figure of merit [6], the improvement is seen through an increase from 0.42 for the cotton paper to 0.45 for the dual-layer system when using a standard *r* value of 7.14 (with assumed peak solar irradiation of 1000 W/m^2^ and blackbody surface sky window emissivity of 140 W/m^2^). Furthermore, nanocellulose-based substrates were characterized to better understand the effects of fiber size and microstructure on the optical characteristics. Finally, by characterizing the relationship between total solar reflectance and dual-layer thickness, ideal top layer thickness can be determined. Thus, it’s possible to optimize the mentioned mechanical and cooling properties and compare to previous works to determine relative efficiency.

## 3 Methodology

### 3.1 BaSO_4_ paint-cotton paper dual-layer thin film sample fabrication

To create the dual-layer thin films, BaSO_4_ nanoparticle-acrylic paint is first made. Lucite International’s Elvacite 2028 acrylic was used for the binder material due to the lower viscosity and ease of use in fabrication. The acrylic powder was slowly added to dimethylformamide (DMF) solvent stirring at 100–200 rpm on a stir plate, with a volume ratio of 1:4 acrylic to DMF. This mixture is then stirred at 200–300 rpm for a minimum of 1 h to allow the acrylic to fully dissolve. It is desired for there to be a 60 % volume concentration of BaSO_4_ nanoparticles and a 40 % volume concentration of acrylic in the final dried product. Using this ratio, BaSO_4_ nanoparticles are added to the acrylic–DMF mixture in small batches and distributed into the mixture using a sonication probe at 30 % amplitude once fully incorporated. Sonication is done for a total of 5 min, with 30 s breaks after 30 s sonication intervals to ensure that the mixture is not overheated past the glass transition temperature of the chosen acrylic, which is reported at 45 °C by Lucite International. After sonication, the paint is immediately used or is used within 5 days of initial synthesis with stirring at 200–300 rpm between use to prevent separation.

**Figure 6:**
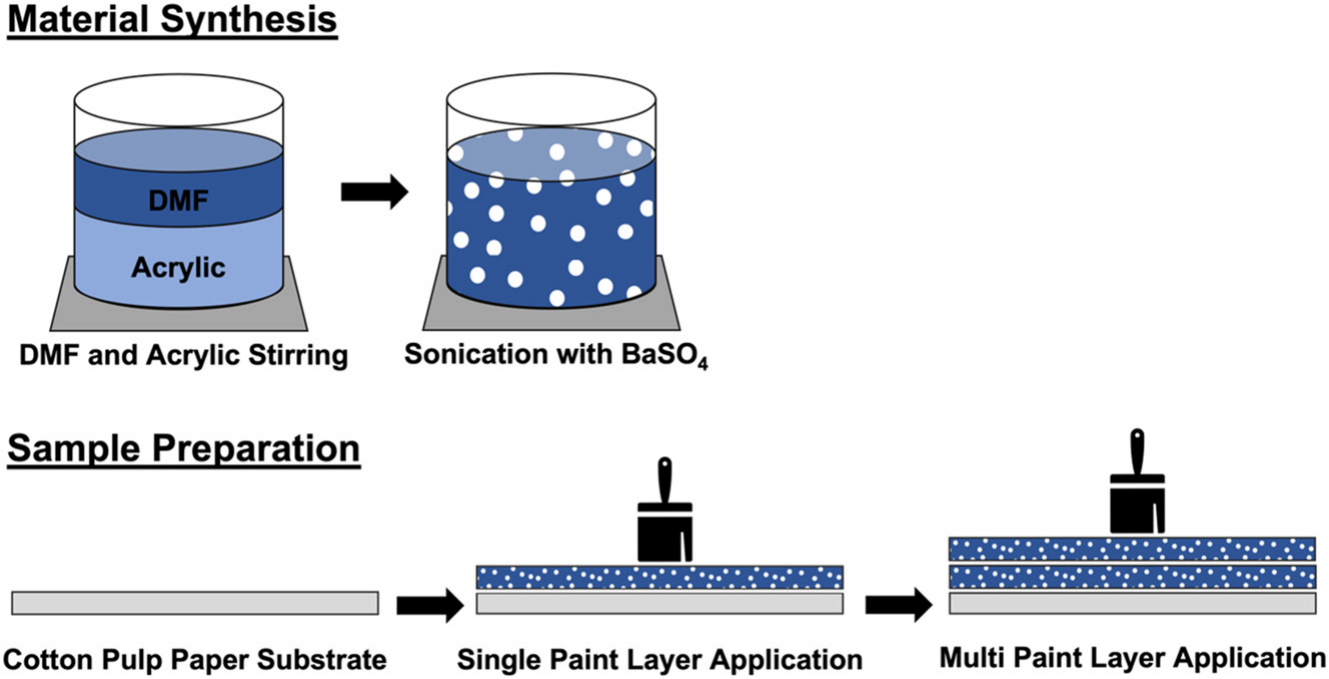
Material synthesis and sample preparation process schematic. For making the paints, dimethylformamide (DMF) solvent and acrylic powder are stirred together on a stir plate. Then, barium sulfate nanoparticles are slowly integrated, and the mixture is sonicated to form the paint. For the sample preparation, brushes or sharp-edged squeegees are used to distribute one or more even layers of paint over the cotton pulp paper substrate. Each layer is allowed to dry between applications.

For the dual-layer film fabrication, a commercial 100 % cotton pulp paper (ProMaster FineArt Inkjet) and cellulose acetate sheet (Hygloss Products Clear Projector Films) were used as the substrates. The paint was used both immediately after sonication as well as a few days later with no differences noticed in final product appearance or performance. Several samples were used for all reported measurements, with varying times of application. The paint was applied using a calibrated block scraper in 20 μm thick wet layers. For greater thicknesses, each layer was allowed to fully dry in fume hood before the next layer was applied. The final dual-layer films were allowed to dry for 4–24 h in a fume hood before use. This range represents how long various samples were left to dry before removed from fume hood and used. This process is visualized in Figure 6. Stated thicknesses are those for the final dry paint layer, which change from the original wet thickness due to the solvent evaporation.

### 3.2 Spectral characterization

The optical properties of the samples were characterized in both UV–Vis-NIR and IR wavelengths using spectrometers. For UV–Vis-NIR characterization, a Perkin Elmer Lambda 950 spectrometer with an integrating sphere was used along with a Spectralon diffuse reflectance standard. The characterization in IR wavelengths was performed on a Nicolet iS50 FTIR spectrometer with a PIKE integrating sphere and a PIKE Technologies diffuse reflectance standard. A stand-alone sample was used for the UV–Vis-NIR and for the mid-IR measurements to avoid contribution of the substrate to emissivity and reflectance. Although a freestanding sample or IR-transparent substrate is preferred for sky window emissivity measurements, this was not successfully attained at this point yet and will be attempted in the future. The spectral reflectance *R*_*λ*_ and transmittance *T*_*λ*_ from 0.25 to 20 μm were measured and quantified. The spectral absorptance *A*_*λ*_ was calculated from measured values by *A*_*λ*_ = 1 − *R*_*λ*_ − *T*_*λ*_. The sky window emissivity was calculated by first obtaining the absorptance at each wavelength from the measured reflectance and transmittance values, and then finding the total absorptance within the sky window, pertaining to 8–13 μm wavelengths.

### 3.3 Total solar reflectance theoretical estimate

Using experimentally obtained solar reflectance, absorptance, and transmittance of single layer paint and cotton paper separately, the overall values can be calculated for the dual-layer films for each paint top layer thickness. This uses the approach presented by Frank Benford using a two-flux approximation for a multi-layered slab [32], [33]. In this way, a theoretical calculation of the overall total solar reflectance for the dual-layer films can be done. As demonstrated in Figure 4, this system can be approximated by considering the total reflection as the summation of the scattering characteristic of the incoming light. As shown in Figure 3(a), let *T*_*i*_ and *R*_*i*_ be the transmittance and reflectance, respectively, of layer *i*, then the total solar reflectance *R*_total_ can be calculated by

$$\begin{array}{c} R_{\text{total}}=R_{1}+T_{A}R_{B}T_{A}+T_{A}R_{B}T_{A}\left(R_{1}R_{B}\right)+T_{A}R_{B}T_{A}\left(R_{1}R_{B}R_{1}R_{B}\right)+\ldots \\ \;=R_{1}+T_{A}R_{B}T_{A}\left(1+\left(R_{1}R_{B}\right)+{\left(R_{1}R_{B}\right)}^{2}+\ldots \right)\\ \;=R_{1}+T_{A}R_{B}T_{A}\left(\frac{1}{1-R_{1}R_{B}}\right) \end{array}$$

Using the predicted total solar optical responses for each layer, the overall total solar reflectance can be calculated.

### 3.4 Outdoor testing

The performance of sample radiative cooling was assessed by concurrently tracking the sample temperature, ambient temperature, and solar irradiation. The outdoor experiment took place in West Lafayette, IN (40.4237°N, 86.9212°W) from November 28th to 30th, 2023. Figure 3a illustrates the outdoor cooling setup, with T-type thermocouples were affixed to the back of the samples, as well as ambient temperature of the testing compartment with a shield from sunlight and convection. To minimize conduction and convection losses while preserving radiative heat exchange, the samples were positioned within an insulated Styrofoam box and covered with a 12 μm PE film along the sides of the compartment. Cooling below ambient was calculated using ambient temperature, as utilizing air temperature in the sample compartment would result in an overestimation, while weather station temperature would lead to an underestimation of cooling capacity. Solar irradiation was tracked with a pyranometer, and data collection occurred at one-minute intervals, with a plotted moving average calculated over 10-min intervals.

## Supplementary Material

Supplementary Material Details
