# Supplementary material for: Efficient radiative cooling of low-cost BaSO4 paint-paper dual-layer thin films
Source: Nanophotonics. 2024 Jan 23;13(5):639–48. doi: 10.1515/nanoph-2023-0642 (PMC11501123; doi:10.1515/nanoph-2023-0642)
Supplement: Supplementary file 1 — Supplementary Material Details [file j_nanoph-2023-0642_suppl_001.docx]

**Supplementary Material**

Efficient Radiative Cooling of Low-Cost BaSO4 Nanoparticle-Paper Dual-Layer Thin Films

Felicelli *et al.*

**Supplementary Figures:**

**
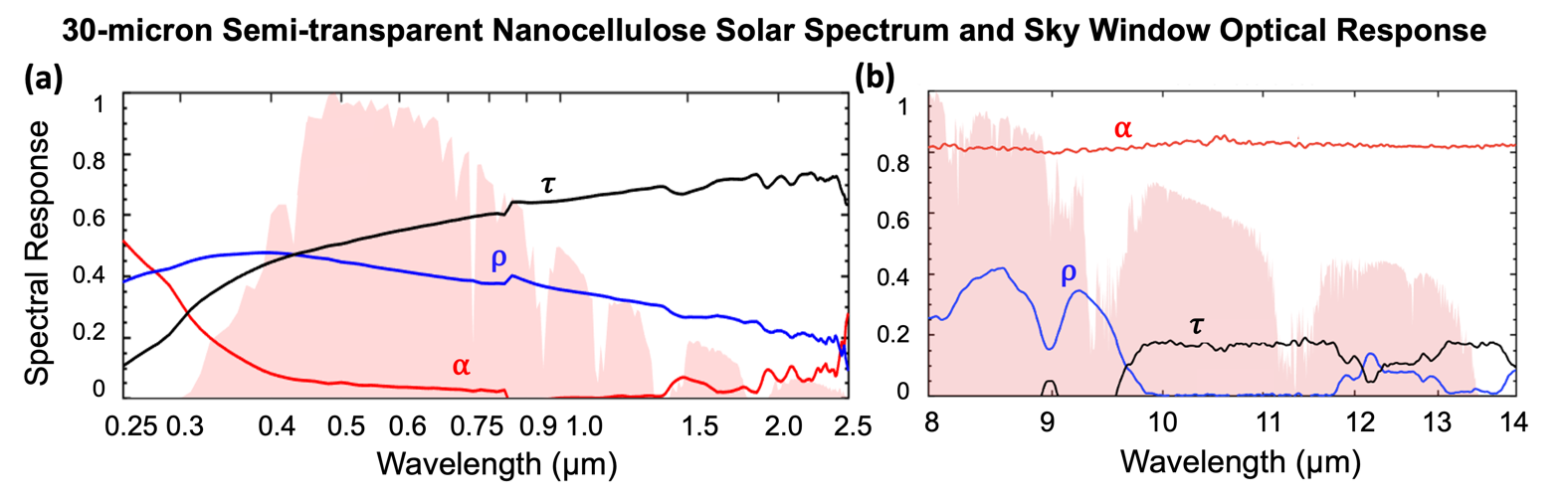
**

**Figure S1.** Example full spectra of semi-transparent nanocellulose, at 33 micron thickness, for solar spectrum and sky window wavelengths. **(a)** Full spectra of solar spectrum reflectance, transmittance, and absorbance of 33 micron thick semi-transparent nanocellulose sample measured on UV-VIS-NIR spectrometer. **(b)** Full spectra of sky window reflectance, transmittance, and absorbance of 33 micron thick semi-transparent nanocellulose sample measured on FT-IR spectrometer.


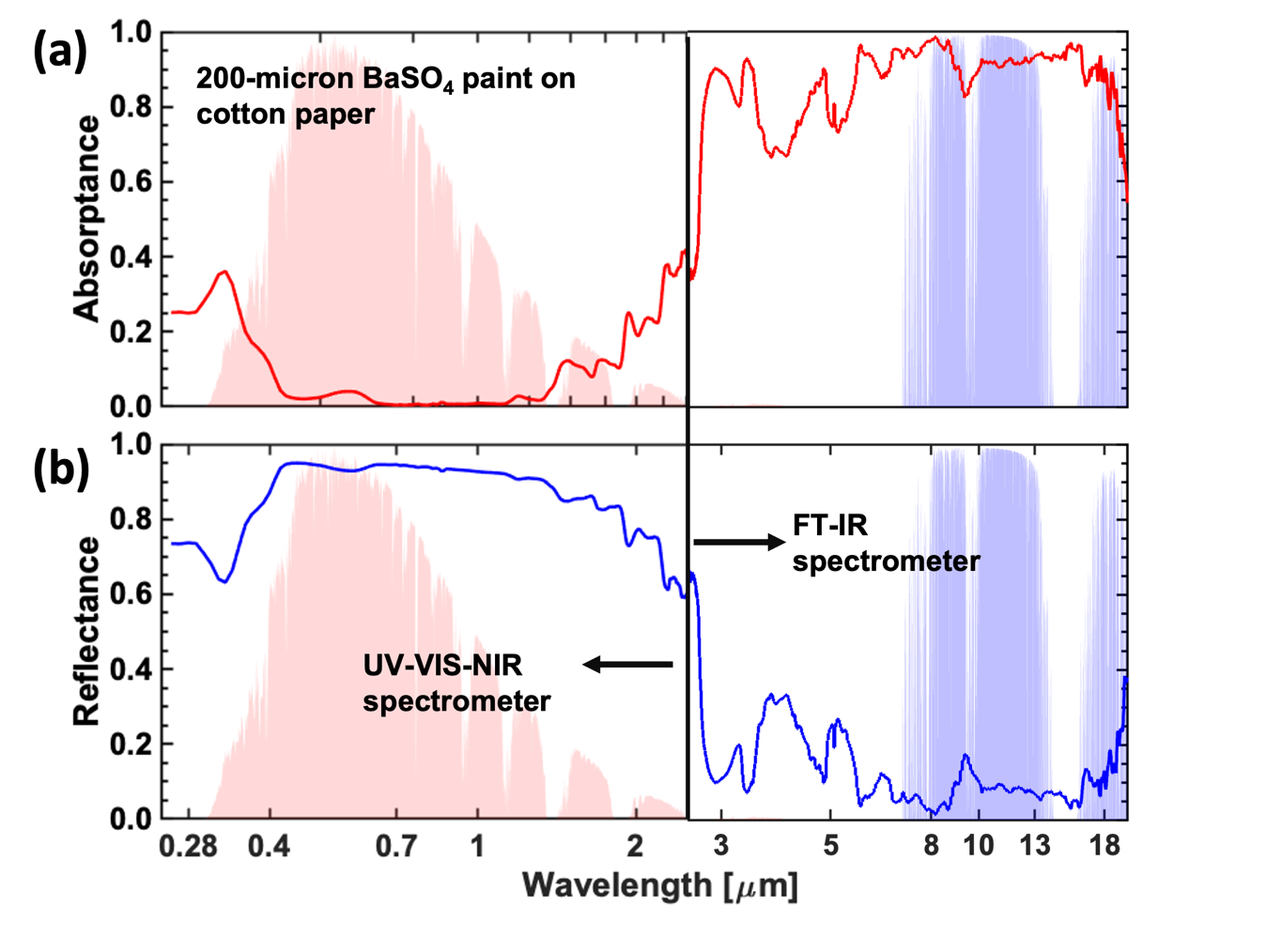


**Figure S2.** Example full spectra of of 200 micron thick layer BaSO4-paint on cotton paper for 0.25-18 micron wavelengths. **(a)** Full spectra of reflectance for BaSO4-paint on cotton paper sample measured on UV-VIS-NIR spectrometer. **(b)** Full spectra absorbance for BaSO4-paint on cotton paper sample measured on FT-IR spectrometer.
